# Structural Exploration on Palmitoyltransferase DHHC3 from *Homo sapiens*

**DOI:** 10.3390/polym14153013

**Published:** 2022-07-26

**Authors:** Meng Tang, Ying Xia, Taoran Xiao, Ruiyu Cao, Yu Cao, Bo Ouyang

**Affiliations:** 1 State Key Laboratory of Molecular Biology, Centre for Excellence in Molecular Cell Science, Shanghai Institute of Biochemistry and Cell Biology, Chinese Academy of Sciences, 320 Yueyang Road, Shanghai 200031, China; tangmeng6@sibcb.ac.cn (M.T.); xiaotaoran2018@sibcb.ac.cn (T.X.); caoruiyu2018@sibcb.ac.cn (R.C.); 2 University of Chinese Academy of Sciences, Beijing 100049, China; 3 Institute of Precision Medicine, The Ninth People’s Hospital, Shanghai Jiao Tong University School of Medicine, 115 Jinzun Road, Shanghai 200125, China; xiaying@shsmu.edu.cn; 4 Department of Orthopaedics, Shanghai Key Laboratory of Orthopaedic Implant, Shanghai Ninth People’s Hospital, Shanghai Jiao Tong University School of Medicine, Shanghai 200011, China

**Keywords:** DHHC3, protein expression and purification, X-ray crystallography, cryogenic electron microscopy

## Abstract

DHHC3 belongs to a family of DHHC palmitoyltransferase, which catalyzes the S-palmitoylation of target proteins by attaching a fatty acyl group to a cysteine. Recently, DHHC3 has been demonstrated to be a promising antitumor target in cancer therapeutics. However, the detailed structure and catalysis mechanism of DHHC3 remain elusive, considering its sequence diversity from the DHHC homologues with known crystal structures. Here, we described the expression and purification of human DHHC3 (hDHHC3) and truncated hDHHC3 with the flexible N-terminal domain (NTD) removed. Purified hDHHC3 proteins were used under various conditions for protein crystallization. LAMTOR1, one of the interacting proteins of hDHHC3 to facilitate the crystallization, was further identified by mass spectrometry and co-immunoprecipitation assay. The structural exploration using cryogenic electronic microscopy (cryo-EM) on the inactive hDHHS3 mutant showed a typical sideview of membrane proteins. These results provide a preliminary guidance for the structural determination of DHHC3.

## 1. Introduction

S-palmitoylation is the most common post-translational protein lipidation in humans. This modification adds a long hydrophobic acyl chain to a cysteine in the target proteins and hence changes their properties, such as the location, structure, stability, and activity [1,2]. In contrast to other kinds of protein lipidation, S-palmitoylation is a reversible modification due to the reversibility of a thioester bond formed between the acyl group and the cysteine residue [3]. More than 3500 human proteins are known to be palmitoylated [4], including neuronal proteins, ion channels, receptors, and scaffold proteins, highlighting the importance of S-palmitoylation in a range of cellular processes [5,6,7,8]. The attachment of fatty acyl groups to cysteine residues is catalyzed by a family of polytopic integral membrane enzymes, called DHHC palmitoyltransferase, owing to their highly conserved Asp-His-His-Cys (DHHC) motif in the catalytic site [9]. So far, at least 23 members of the DHHC family named DHHC1 to DHHC24 have been identified since the first discovery in the early 2000s [9,10,11,12]. These enzymes use fatty acyl-CoA as the ubiquitous fatty acyl donor to target different substrate proteins; some enzymes have a broad range of target proteins, while some are highly specific [13].

Among the discovered DHHC enzymes, DHHC3 has attracted a great interest as an antitumor target in cancer therapeutics. DHHC3 is closely related to many cancers: elevated expression levels of DHHC3 were found in patients with prostate, kidney, brain, colorectal, breast cancers, etc. [14]. A high expression of DHHC3 was also reported to be associated with the reduced survival rate of many cancer patients. Recently, DHHC3 was identified as the palmitoyltransferase [15] for PD-L1, which is a transmembrane protein highly expressed in cancer cells for immune evasion [16]. The palmitoylation of residue C272 in PD-L1 by DHHC3 promotes PD-L1 stability and increases PD-L1 levels on the plasma membrane (PM) [15]. Moreover, DHHC3-palmitoylated CMTM6 can further stabilize PD-L1 in the PM and recycling endosomes and thereby prevent PD-L1 degradation [17,18]. In addition, DHHC3 can palmitoylate integrin α6 and β4 subunits (α6β4) [19] and phosphatidylinositol 4-kinase II (PI4KIIα) [20], and plenty of substrate proteins are strongly associated with oxidative stress [21]. Therefore, the inhibition of DHHC3 would lead to an indirect inhibition of tumor growth and development, which provides an alternative anti-cancer approach and raises an urgent need for the structural determination of DHHC3 for the guidance of specific inhibitor design [22]. 

The structures of two DHHC enzymes, human DHHC20 (hDHHC20) and zebrafish DHHC15 with a catalytically inactive mutation C153S (zfDHHS15), were recently determined by X-ray crystallography [23]. Both hDHHC20 and zfDHHS15 contain four transmembrane helices with a tepee-like arrangement. The cysteine-rich domain (CRD) containing the key catalytic DHHC motif projects into the cytosol. The DHHC-CRD also contains a CCHC zinc-finger domain, and two Zn^2+^ ions bind to the conserved cysteines and histidines. The crystal structure of a complex formed by hDHHC20 with its inhibitor 2-bromopalmitate (2-BP) to mimic the acyl intermediate was further solved, showing that the acyl chain is inserted into an enzyme cavity composed of the four TM helices within the membrane bilayer, in which TM3 contributes the most in contact with the acyl chain [23]. They further reported a structure of a catalytically inactive mutant hDHHS20 in a complex with palmitoyl-CoA and elucidated that the polar and ionic interactions between the CoA headgroup and the cytosolic domain are also important for the substrate binding [24]. 

DHHC3 contains 299 amino acids and shares a high sequence identity from 97.3% to 100% among all mammals (Figure S1 and Table S1). However, the sequence of human DHHC3 (hDHHC3) is very different from zfDHHC15 and hDHHC20 with the sequence identities of 25.4% and 29.5% (Figure S2), respectively. Moreover, DHHC3 can palmitoylate a broader range of target proteins than DHHC15 and DHHC20 [8], indicating that DHHC3 may have different structural features from the known architectures of the DHHC enzymes. To gain insight into the substrate selection and catalytic mechanism of DHHC3, here, we explored the structural characterization of hDHHC3 using X-ray crystallography and cryogenic electron microscopy (cryo-EM). We found that the flexible N-terminal domain (NTD) of hDHHC3 was easily degraded during the purification, while NTD-truncated hDHHC3s remain the auto-palmitoylation activity. Mass spectrometry identification of the interacting proteins of hDHHC3 revealed that proteins such as LAMTOR1 can be pulled down together with hDHHC3. Cryo-EM studies of the inactive hDHHS3 mutant showed a typical sideview of transmembrane proteins with an extra-membranous domain protruding out from the ellipsoidal micelles. 

## 2. Materials and Methods

### 2.1. Materials

PrimeSTAR DNA Polymerase (Cat. R040) was purchased from Takara (Shiga, Japan). Sgs I (Cat. FD1894) and Not I (Cat. FD0593) restriction enzymes and T4 DNA ligase (Cat. EL0012) were purchased form Thermo Fisher (Waltham, MA, USA). A transfecting grade DNA magnetic purification kit (Cat. GO-ERPS) was from Geneon BioTech (Changchun, China). Cellfectin II (Cat. 10362100) was purchased from Invitrogen (Waltham, MA, USA). Insect cell medium ESF 921 (Cat. 96-001) was purchased from Expression System (Davis, CA, USA). Strep-Tactin sepharose resin (Cat. 2-1201-010) was purchased from IBA (Goettingen, Germany). n-Dodecyl-β-D-Maltoside (DDM) (Cat. D310), n-Undecyl-β-D-Maltoside (UM) (Cat. U300), n-Decyl-β-D-Maltoside (DM) (Cat. D322), n-Nonyl-β-D-Maltoside (NM) (Cat. N330), n-octyl-β-D-glucopyranoside (OG) (Cat. O311), 2,2-didecylpropane-1,3-bis-β-D-maltopyranoside (LMNG) (Cat. NG310) and glyco-diosgenin (GDN) (Cat. GDN101) were purchased from Anatrace (Maumee, OH, USA). Lauryldimethylamine-N-Oxide (LDAO) (Cat. 40236) was purchased from Sigma (Darmstadt, Germany). Superdex 200 Increase 10/300 GL column (Cat. 28990944) was purchased from GE healthcare (Atlanta, GA, USA). 2-Bromopalmitic acid (2-BP) (Cat. 21604) was purchased from Sigma. X-tremeGENE HP DNA Transfection Reagent (Cat. 06-366-236-001) was purchased from Roche (Basel, Switzerland). HA affinity Gel (Cat. GNI4510-HA) was purchased from GNI (Tokyo, Japan). HA tag Rabbit mAB (Cat. 3724) was purchased from CST (Danvers, MA, USA). Anti-LAMTOR1 Rabbit pAB (Cat. Ab121157) and Goat anti rabbit IgG (H+L) conjugated HRP (Cat. Ab97051) were purchased from Abcam (Boston, MA, USA).

### 2.2. Methods

#### 2.2.1. Construct Design and Cloning 

The hDHHC3 gene was a gift from Dr. Jie Xu (Institutes of Biomedical Sciences, Fudan University, Shanghai, China). The hDHHC3 gene was amplified by polymerase chain reaction (PCR) (30 cycles of 10 s at 95 °C, 5 s at 55 °C, 90 s at 72 °C). The genes were digested by Sgs I and Not I restriction enzymes and ligated into a pFastBac-Dual plasmid with the digestion of Sgs I and Not I. The pFastBac-Dual plasmid was modified to contain a Twin-strep tag or a 10 × His tag at the 3′-end of the multiple cloning site (MCS) at downstream of the polyhedrin (PH) promoter. The resulting recombinant plasmids were transformed into *Escherichia coli* (*E. coli)* strain DH5α. Ampicillin-resistant transformants were selected and then confirmed by DNA sequencing. 

For N-terminal truncated (Δ10, Δ21, Δ30, Δ35, and Δ40) and inactive (C157S) hDHHC3 mutants, the mutations were introduced by PCR using primers for truncated or mutagenic constructs. Amplified DNAs were transformed into DH5α with the ampicillin selection. All constructs were further confirmed by DNA sequencing.

#### 2.2.2. Producing Bacmid and Baculovirus

Approximately 300 ng pFastBac-Dual plasmids containing the hDHHC3 gene (or mutants) were transformed into *E. coli* DH10Bac competent cells and cultured for 5 h to produce a recombinant bacmid. The positive clones were selected using a blue–white selection method and further verified by clonal PCR using the M13 primers (Forward: 5′-CCCAGTCACGACGTTGTAAAACG-3′; Reverse: 5′-AGCGGATAACAATTTCACACAGG-3′). Then, bacmids were extracted and purified using a magnetic purification kit.

For recombinant baculovirus production, around 1.0 × 10^6^ Sf9 insect cells were seeded into a 3.5 cm dish with 2 mL of ESF 921 media and transfected with purified bacmids using Cellfectin II reagents and cultured at 27 °C for 4–5 days. The Sf9 cell culture was harvested and centrifuged at 500× *g* at room temperature for 5 min. The supernatant containing recombinant baculoviruses was added into 50 mL Sf9 cells at a density of around 1.5 × 10^6^ cells/mL. Infected cells were then cultured for 3 days to produce more baculoviruses for large-scale protein expression. Recombinant baculoviruses were stored at 4 °C, avoiding light.

#### 2.2.3. Recombinant Protein Expression and Purification

The recombinant baculoviruses were added into High Five cells at 2.5–3.0 × 10^6^ cells/mL to reach a final ratio of virus to cell culture 1:50 (*v*/*v*). Infected High Five cells were cultured at 27 °C gently shaking at 130 rpm for 48–60 h until the viability of High Five cells was lower than 80%. High Five cells were collected by centrifugation at 1500× *g* at room temperature for 15 min. Cell pellets were frozen by liquid nitrogen and stored at −80 °C.

For crystallization study, hDHHC3 or mutants were extracted by n-Dodecyl-β-D-Maltoside (DDM). Then, 1 L of the High Five cells expressing recombinant hDHHC3 proteins were resuspended in 50 mL of lysis buffer (150 mM NaCl, 10% glycerol, 1 mM TCEP, 5 mM MgCl_2_, 10 μg/mL DNase I, 1 mM PMSF and cocktail, 25 mM HEPES pH 7.5); then, they were disrupted by a high-pressure homogenizer (300 bar) at 4 °C. A low-speed (4000× *g*) centrifugation was applied to remove cell debris for 10 min at 4 °C. The supernatant was separated by ultracentrifugation at 150,000× *g* for 1 h. The membrane fraction was solubilized in 1.5% DDM (*w*/*v*) buffer (150 mM NaCl, 10% glycerol, 1.5% DDM, 1 mM iodoacetamide, 25 mM HEPES pH 7.5) at 4 °C for 2 h. After a further step of 45,000× *g* centrifugation at 4 °C for 45 min, the supernatant was collected and incubated with 2 mL Strep-Tactin sepharose resin pre-equilibrated by buffer W (150 mM NaCl, 10% glycerol, 2 mM DDM, 25 mM HEPES pH 7.5) at 4 °C for 1 h. The resin was then rinsed with 20 column volume (CV) of buffer W and eluted with 3 CV of buffer E (150 mM NaCl, 10% glycerol, 2 mM DDM, 4 mM desthiobiotin, 25 mM HEPES pH 7.5). The protein was concentrated to ≈1 mg/mL using a 30 kDa cut-off concentration tube (Millipore, Darmstadt, Germany). Protein samples were then injected into a Superdex 200 Increase 10/300 column (GE Healthcare) and eluted with buffer F (150 mM NaCl, 1 mM TCEP, 1 mM DDM, 25 mM HEPES pH 7.5). The peak fractions were collected and analyzed by SDS-PAGE. Typically, 0.5–0.7 mg hDHHC3 (or hDHHC3 variants) with a Twin-strep tag can be obtained from 1 L High Five cells. Afterwards, 0.5 mM 2-bromopalmitic acid (2-BP) were added into the buffers used during the purification for crystallization trials with inhibitors.

For coupled enzyme assay, iodoacetamide was removed during the protein purification to avoid cysteine blocking. Instead, 1 mM TCEP was added in buffers to maintain reductive environment through protein purification steps.

For cryo-EM study, hDHHS3 was extracted as described before except using LMNG instead of DDM. The membrane fraction was resuspended in 1% LMNG (*w*/*v*) buffer (150 mM NaCl, 10% glycerol, 1% LMNG, 1 mM TCEP, and 25 mM HEPES pH 7.5) at 4 °C for 2 h. After a 45,000× *g* centrifugation step at 4 °C for 45 min, the supernatant was collected and incubated with 2 mL of Strep-Tactin sepharose resin at 4 °C for 1 h. The resin was then rinsed with 10 CV of buffer G (150 mM NaCl, 10% glycerol, 0.04% GDN, 1 mM TCEP, 25 mM HEPES pH 7.5) and eluted with 3 CV of buffer E (150 mM NaCl, 10% glycerol, 0.04% GDN, 1 mM TCEP and 4 mM desthiobiotin, 25 mM HEPES pH 7.5). The protein was concentrated using a 30 kDa cut-off concentration tube (Millipore) to ≈1 mg/mL. Following, size exclusion chromatography was carried out using a Superdex 200 Increase 10/300 column (GE Healthcare) in 150 mM NaCl, 1 mM TCEP, 0.02% GDN, and 25 mM HEPES pH 7.5. The peak fractions were collected and concentrated to ≈2 mg/mL.

#### 2.2.4. SDS-PAGE and Western Blot

All protein samples were analyzed by 12% (*w*/*v*) SDS-PAGE. For Western blot, protein samples were loaded to a SDS-PAGE gel and transferred to PVDF membranes. The blots were incubated in 5% (*w*/*v*) skim milk–TBST solution at room temperature for 2 h and followed by incubation in the antibody solution (5% BSA in TBST with antibody) at 4 °C overnight. Blots were then incubated in HRP conjugated antibody solution (1:10,000 *v/v* in TBST) at room temperature for 1 h. The detection of C-terminal Strep-tagged hDHHC3s was performed using Strep-Tactin HRP conjugates (1:5000 *v*/*v*). The detection of N-terminal HA-tagged hDHHC3s was performed using anti-HA tag antibody (1:1000 *v*/*v*). The detection of C-terminal GFP-tagged LAMTOR1 was performed using anti-LAMTOR1 antibody (1:1000 *v*/*v*). Chemiluminescence HRP substrate was used to develop chemiluminescence and visualized using Bio-Rad ChemiDoc MP.

#### 2.2.5. Coupled Enzyme Assay

The auto-palmitoylation activity of hDHHC3 and its truncated variants was measured using a coupled-enzyme assay carried out at 30 °C in a 96-well plate. For each reaction, 1 μg hDHHC3 proteins were mixed with 0.25 μL 100 mM DTT, 16 mU α-KDH, 25 μL 4 × reaction buffer (1 mM EDTA, 8 mM α-ketoglutaric acid, 1 mM NAD^+^, 0.8 mM thiamine pyrophosphate, 200 mM sodium phosphate pH 7.2), and H_2_O was added to reach a volume of 50 μL. Then, 50 μL palmitoyl-CoA with different concentrations was added into the reaction mixture. The fluorescence of the product NADH was monitored for 30 min (340 nm excitation/465 nm emission) and recorded by a SpectraMax M5 microplate reader (Molecular Devices, San Jose, CA, USA). The linear range of the reaction curve was used to determine the initial velocity. XY plots of velocity vs. concentration of palmitoy-CoA were fitted to the Michaelis-Menten equation using non-linear least square fitting (Graphpad Prism 8). K_m_ and V_max_ were estimated based on the Michaelis-Menten model fitting. K_cat_ was determined by dividing V_max_ by the enzyme concentration. K_m_ and K_cat_ values are the average of three repeated measurements. *P*-values were calculated using two-tailed Student’s *t* tests.

#### 2.2.6. MALDI-TOF Mass Spectrometry 

For molecular weight verification, 1 μL diluted protein samples purified by size exclusion chromatography (SEC) were mixed with 1 µL sinapic acid (SA) matrix; then, they were spotted immediately on the MALDI chips and examined by 5800 MALDI-TOF/TOF (Applied Biosystems, Waltham, MA, USA). Further dilution using SA at the ratio of 1:10 to 1:100 (*v*:*v*) may be applied until the samples can be accurately measured.

#### 2.2.7. Detergent Exchange for Screening

Purified hDHHC3-Δ21 was further exchanged into different detergents using size exclusion chromatography. The protein sample was concentrated to ≈0.5 mg/mL and then loaded onto a Superdex 200 Increase 10/300 column in the buffer: 150 mM NaCl, 25 mM HEPES pH 7.5 with different detergents. Detergent concentrations used for SEC are at least 2-fold of their corresponding critical micelle concentration (CMC), which are listed below: DDM, 1 mM; n-Undecyl-β-D-Maltoside (UM), 2 mM; n-Decyl-β-D-Maltoside (DM), 4 mM; n-Nonyl-β-D-Maltoside (NM), 12 mM; n-octyl-β-D-glucopyranoside (OG), 40 mM; Lauryldimethylamine-N-Oxide (LDAO), 0.15% (*w*/*v*).

#### 2.2.8. Protein Crystallization

Sitting-drop vapor diffusion crystallization trials were set up on 96-well sitting drop plastic plates (SWISSCI, Buckinghamshire, England) and performed with Gryphon LCP (ARI, Sunnyvale, CA, USA). The protein concentration is ≈10 mg/mL. For each well, 0.2 μL protein samples were mixed with 0.2 μL mother liquid. Crystallization trials were then plated at 4 °C and monitored for 35 days.

For lipidic cubic phase (LCP) crystallization, 15–20 mg/mL proteins were incorporated into molten monoolein at a volume ratio of 2:3. Protein solution and molten monoolein were mixed by a syringe coupler until the mixture became homogenous and transparent. LCP crystallization trials were set up on glass sandwich plates (FAstal Biotech, Shanghai, China) using Gryphon LCP. For each well, 50 nL LCP samples were covered by 1.2 μL mother liquid. Crystallization trials were then plated at room temperature and monitored for 35 days.

#### 2.2.9. Pull Down Assay of hDHHC3-Interacting Proteins

The C-terminal Strep-tagged hDHHC3 was expressed using High Five cells and purified using Strep-Tactin affinity chromatography as described before. Strep-Tactin sepharose beads were used as a negative control. HEK 293T cells were cultured in a 60 mm dish. Cells were collected and lysed by RIPA solution at 4 °C for 30 min. The cell lysate was centrifuged at 16,500× *g* at 4 °C for 20 min to remove cell debris. The HEK 293T cell lysate was incubated with Strep-Tactin resin with or without hDHHC3 at 4 °C for 30 min. Subsequently, the beads were washed with the buffer W to remove the unbound proteins. Protein solutions were eluted by buffer E (150 mM NaCl, 1 mM DDM, 4 mM desthiobiotin, 1 mM TCEP, 25 mM HEPES pH 7.5).

#### 2.2.10. LC-MS/MS

The protein samples eluted from Strep-Tactin affinity resin were subjected to in-solution digestion. The pH value of the protein and control samples was adjusted to 8.0 by adding 500 mM Tris-HCl (pH 8.0), which was followed by reduction in 10 mM DTT for 30 min and alkylation in 30 mM iodoacetamide for 30 min in the dark. Five-fold volumes of chilled acetone were added into the samples for protein precipitation. The protein pellets were resuspended in 20 μL of 25 mM ammonium bicarbonate and then digested overnight with 1:50 (*w*/*w*) MS-grade trypsin at 37 °C. After desalting with self-parked StageTips, the peptide samples were separated on a self-packed column (75 μm id, 15 cm, C18-AQ, 1.9 μm, Dr Maisch) by an EASY-nLC 1200 (Thermo Fisher Scientific, Waltham, MA, USA) at a flow rate of 250 nL/min. The eluates were directly analyzed by a Q-Exactive HF instrument (Thermo Fisher Scientific, Waltham, MA, USA) using a data-dependent acquisition mode. The raw data were searched against the database using Proteome Discoverer 2.2 (Thermo Fisher Scientific, Waltham, MA, USA). The decoy database searches were also performed in parallel, and peptides and proteins less than 1% false discovery rate (FDR) were accepted.

#### 2.2.11. Co-Immunoprecipitation

HEK 293T cells were cultured in 60 mm dishes until 70–80% confluency. N-terminal HA tagged hDHHC3 and C-terminal GFP-tagged LAMTOR1 were transfected using DNA transfection reagents. The cells were collected and lysed by RIPA cell lysis buffer with 1 × protease inhibitor cocktail. Cell lysate was incubated with HA affinity resin at 4 °C overnight and then rinsed for three times by phosphate-buffered saline and 0.05% Tween-20 (PBST); HA affinity resin was added with 20 μL 2 × SDS-PAGE loading buffer and boiled for 5 min at 95 °C. Cell lysate and immunoprecipitation samples were then performed by Western blot.

#### 2.2.12. Cryo-EM Sample Preparation, Data Collection and Processing

A glow-discharged holey carbon grid (Quantifoil R1.2/1.3 Au, 300 mesh) was used for cryo-grid sample preparation. Samples were vitrified using Vitrobot Mark IV (FEI) and operated at 8 °C and 100% humidity. Three microliter aliquots of hDHHS3 protein sample were applied to the grids and incubated for 10 s. The grids were then blotted for 1.5 s and rapidly plunged into liquid ethane cooled by liquid nitrogen. The dataset was collected with EPU software (FEI) using 200 kV Talos Arctica (FEI) equipped with a Falcon III detector (Thermo Fisher Scientific, Waltham, MA, USA). The data were collected at a nominal magnification of ×120,000 (corresponding to a physical pixel size of 1.24 Å), with a defocus range between −1.0 and −2.5 μm. The dose rate was set to 0.59 electrons/Å^2^/s, and the total exposure time was 68 s, resulting in a total dose of 40 electrons/Å^2^, which was fractionated into 32 frames. A total of 439 cryo-EM images were collected, and motion correction was performed on the dose-fractioned image stacks using MotionCor2 with dose weighting. The contrast transfer function (CTF) parameters of each image were determined with Gctf, and 133,837 particles were picked using Gautomatch v0.56. Subsequent image processing steps were performed with RELION 3. The particles were first extracted with 4 × binning, and junk particles were removed by two rounds of 2D classifications. Then, 33,604 particles were extracted with 2 × binning, and junk particles were removed by two rounds of 2D classifications. At last, 25,149 particles remained.

## 3. Results

### 3.1. Expression and Purification of Full Length DHHC3

We first cloned hDHHC3 with a C-terminal Twin-Strep tag or with a C-terminal 10 × His tag and tested the recombinant expression using the Bac to Bac expression system in insect cells. The optimal production was obtained using the baculovirus system in High Five cells with the Twin-Strep tag. Expressed hDHHC3 was then solubilized and purified using the common mild detergent for membrane proteins, DDM. 

**Figure 1:**
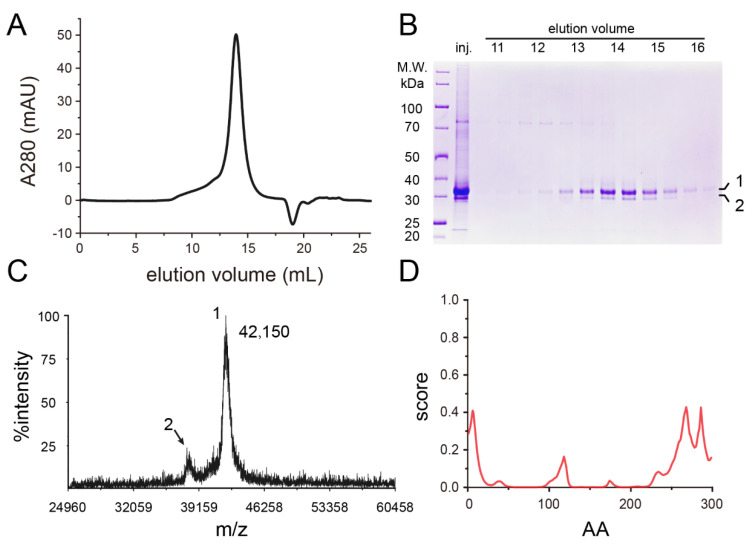
Purification and characterization of hDHHC3. (**A**) Representative size exclusion chromatography profile of full-length hDHHC3; (**B**) SDS-PAGE analysis of hDHHC3. Two bands are shown in the gel with the predicted size (band 1) and the degraded (band 2); (**C**) MALDI-TOF MS result of hDHHC3. The main peak (peak 1) shows that the molecular weight of the hDHHC3 protein is 42,150 Da, while the minor peak (peak 2) at ≈38 kDa represents the degraded hDHHC3; (**D**) The disordered regions of hDHHC3 were predicted using IUPred2.

Since hDHHC3 contains 16 cysteines, iodoacetamide was introduced to block the possible disulfide bond formation between cysteine residues during the purification. Iodoacetamide is a widely used cysteine modification compound in the membrane protein purification and crystallization [25,26]. The protein purification started with Strep-Tactin affinity chromatography, which was followed by size exclusion chromatography using a Superdex 200 Increase 10/300 column (Figure 1A). The protein purity of the purified recombinant protein was examined by SDS-PAGE (Figure 1B). An extra minor band was observed below the major protein band with a predicted monomeric protein size. MALDI-TOF mass spectrometry (MS) was performed to confirm the production of hDHHC3 (Figure 1C). Considering the modification of iodoacetamide on hDHHC3 that adds a carbamidomethyl group (57.07 Da) to exposed cysteine residues, the theoretical molecular weight of hDHHC3 is 42,243 Da, if all the cysteines are modified by iodoacetamide. The experimental molecular weight is 42,150 Da, which is close to the theoretical value. The MS results also showed a peak at ≈38 kDa, indicating the existence of a degraded protein. We then used the IUPred2 program [27] to predict the disordered region of hDHHC3 (Figure 1D). The disordering scores of 5–33 residues in the N-terminus and 283–299 residues in the C-terminus are much higher than that of other residues. Given the fact that the recombinant hDHHC3 protein is C-terminal tagged and successfully purified, the degradation is likely happening at the N-terminus of hDHHC3.

### 3.2. Expression and Purification of Truncated DHHC3

**Figure 2:**
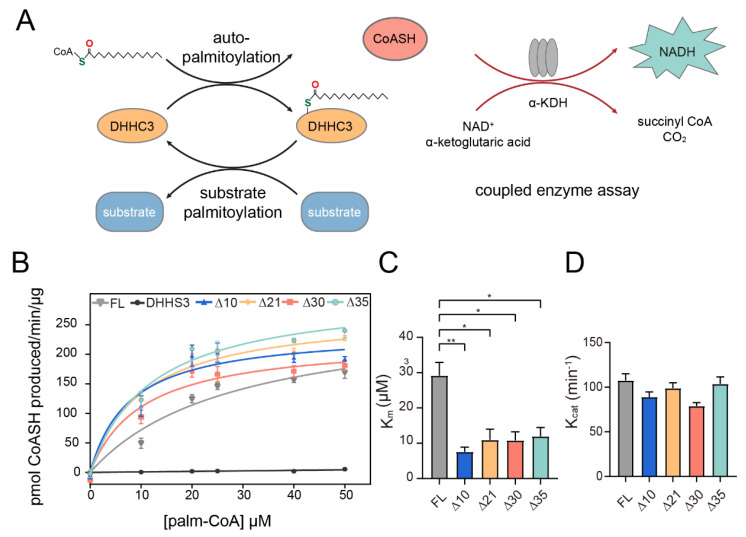
Functional characterization of full-length and truncated hDHHC3s. (**A**) Schematic illustration of Ping-Pang kinetic mechanism of DHHC3 mediated palmitoylation and its coupled enzyme assay. (**B**) The results from the coupled enzyme assay for full-length hDHHC3, inactive hDHHS3 and N-terminal truncated hDHHC3, respectively. Data were fitted into a Michaelis–Menten equation shown as mean ± SEM of 3 repeated measurements. (**C**) K_m_ and (**D**) K_cat_ values of full-length and truncated hDHHC3 determined from (**B**). Data are shown as mean ± SEM of 3 repeated measurements, and *p*-values were calculated using two-tailed Student’s *t* tests. *, *p* < 0.05; **, *p* < 0.01.

To rule out the heterogeneity influence of the degraded proteins, several N-terminal truncated hDHHC3s were constructed and expressed, including Δ10, Δ21, Δ30, and Δ35, indicating that the first 10, 21, 30, and 35 residues of hDHHC3 were truncated, respectively (Figure S3A). In addition, Δ40 was constructed to remove the whole N-terminal region right before the transmembrane domains. The SDS-PAGE analysis of these truncated proteins showed that hDHHC3-Δ40 was not expressed. Meanwhile, only hDHHC3-Δ10 still exhibited some degraded proteins (Figure S3B), and other constructs showed no degradation after the truncation. hDHHC3-Δ30 showed much lower expression, which was not selected for the further purification and crystallization screening, since a large amount of proteins are required for growing crystals. hDHHC3-Δ21 and hDHHC3-Δ35 gave comparable yields (Figure S3C,D) as full-length hDHHC3 following the purification protocol described above, which was used for further structural investigation. In order to check whether these truncated proteins maintain enzymatic activity, a coupled enzyme assay was performed on these truncated hDHHC3s to characterize their auto-palmitoylation ability (Figure 2A), in which free CoASH generated from hDHHC3 auto-palmitoylation was indirectly monitored by the production of reduced nicotinamide adenine dinucleotide (NADH) in a coupled enzymatic reaction [23]. The results from the coupled enzyme assays showed that all the truncated hDHHC3s maintain auto-palmitoylation activity (Figure 2B), while the C157S mutant displayed no enzymatic activity. However, truncated hDHHC3s showed smaller K_m_ values (Figure 2C), indicating a stronger binding affinity to palmitoyl-CoA than that of wild type, while the K_cat_ values are similar (Figure 2D), indicating similar maximum turnover rates between them. 

### 3.3. Crystallization of hDHHC3

Based on the above purification and characterization results of the truncated proteins, full-length hDHHC3, hDHHC3-Δ21, and hDHHC3-Δ35 were tested in the crystallization studies. The sitting-drop vapor diffusion method and LCP crystallization method were carried out, respectively, using the commercial crystallization kits in 96-well plates, including Index, Crystal Screen, Crystal Screen 2, MembFac (from Hampton Research, Aliso Viejo, CA, USA), MemGold, MemGold2, MemStart, MemSys, MemChannel, MemTrans, PGA (from Molecular Dimensions, Maumee, OH, USA) and WIZARD classic (from Rigaku, Tokyo, Japan). The crystallization trials were also performed for hDHHC3 or truncated hDHHC3s in the presence of inhibitor 2-BP. In addition, all of the above crystallization screening was executed on hDHHC3 or truncated hDHHC3s with C157S mutation. Unfortunately, no crystals grew in any of the crystallization trials. Heavy precipitation was observed in the presence and absence of 2-BP.

We then examined the effects of different detergents on the crystallization. hDHHC3-Δ21 was tested and exchanged into different detergents by SEC, including 1 mM DDM, 2 mM UM, 4 mM DM, 12 mM NM, 40 mM OG, and 0.15% LDAO. These detergents are commonly used in the membrane protein crystallization. hDHHC3-Δ21 shows high stability in DDM, UM, DM, and LDAO micelles with homogenous and sharp elution peaks, while the SEC file showed that hDHHC3-Δ21 in NM and OG micelles is inhomogeneous, and a large amount of proteins disappeared during this purification step, indicating that hDHHC3-Δ21 is not stable in NM and OG micelles (Figure S4). Usually, membrane proteins in shorter acyl chain detergents lead to a tighter packing in crystallization. We then selected DM to screen the crystallization conditions as mentioned above due to its shorter acyl chain. No crystals appeared in any crystallization experiments.

Next, we referred to identify the interacting proteins of hDHHC3 to facilitate the crystallization, which is another common method to obtain the crystals. A pull-down assay combined with LC-MS/MS identification was performed. More than 21 proteins were identified by mass spectrometry based on the PSM values and Sum PEP scores. Among them, 15 proteins were detected repeatedly in three identification experiments (Table 1), in which syntaxin-12, protein disulfide-isomerase TMX3 (TMX3), protein jagunal homolog 1 (JAGN1), sodium/potassium-transporting ATPase subunit beta-3 (ATP1B3), adipocyte plasma membrane-associated protein (APMAP) proteins, and ragulator complex protein LAMTOR1 (LAMTOR1) have the potential to colocalize with hDHHC3, as their respective subcellular localizations are the plasma membrane, endoplasmic reticulum (ER), Golgi apparatus, endosomes, or lysosomes, where hDHHC3 specifically localizes [28,29]. 

**Figure 3:**
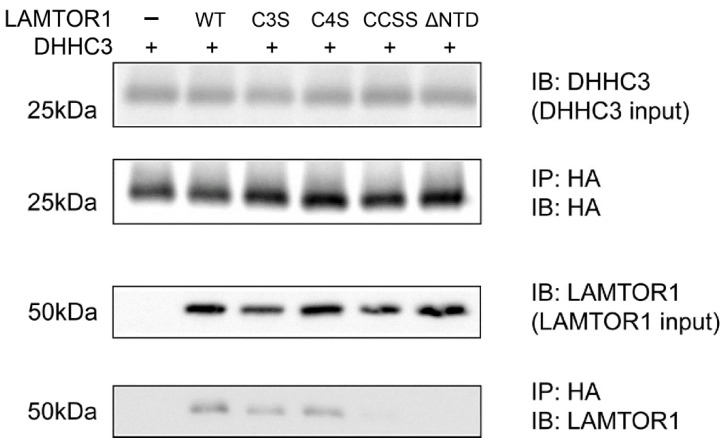
Pull-down assay and validation of interactions between hDHHC3 and LAMTOR1 by Western blot. hDHHC3s and LAMTOR1 were co-transfected into HEK 293T cells, subjected to co-immunoprecipitation (Co-IP) and analyzed by Western blot. The input proteins were analyzed by immunoblotting (IB) using total cell extracts (top and third panels). Co-immunoprecipitated hDHHC3 proteins using anti-HA affinity resin were probed with anti-HA (second panel) and anti-LAMTOR1 antibody (bottom panel), respectively.

Since LAMTOR1 palmitoylation was previously reported [30], indicating its ability to interact with DHHC enzymes, in vitro co-immunoprecipitation (Co-IP) experiments were further carried out on LAMTOR1 to verify the interactions between hDHHC3 and LAMTOR1. N-terminal HA tagged hDHHC3 and C-terminal GFP tagged LAMTOR1 were co-transfected into HEK 293T cells, and Western blot analysis showed that both hDHHC3 and LAMTOR1 were detected in the IP product (Figure 3). We mutated or deleted the two cysteines in LAMTOR1 (C3, C4) to validate the interactions between hDHHC3 and LAMTOR1. Double mutations and removal of the NTD of LAMTOR1 containing the cysteines blocked the protein–protein interactions, while the single mutants (C3S, C4S) still maintained the binding to hDHHC3. However, LAMTOR1 was not expressed successfully in insect cell expression systems, and thereby, no further structural investigation was performed using LAMTOR1 as a co-crystallization helper.

### 3.4. Cryo-EM Study of DHHC3

**Figure 4:**
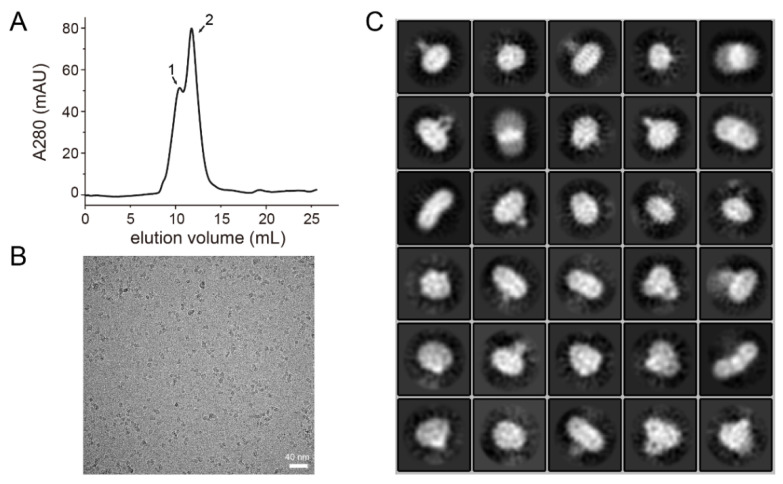
Characterization of hDHHS3-Δ35 by 200 kV cryo-EM measurements. (**A**) Representative size-exclusion chromatography profile of hDHHS3-Δ35 purified in GND micelles. At least two states were detected in SEC, possibly referring to hDHHS3-Δ35 dimers (peak 1) and hDHHS3-Δ35 monomers (peak 2). (**B**) A representative micrograph of the hDHHS3-Δ35 protein sample. (**C**) Two-dimensional (2D) class averages of hDHHS3-Δ35 in GDN micelles.

During the protein preparation, we noticed that the C157S mutant of hDHHC3-Δ35 (hDHHS3-Δ35) showed an obvious dimeric band at ≈50 kDa (Figure S5A), which gave a chance for us to determine the structure of hDHHS3-Δ35 using electronic microscopy, since recent cryo-EM studies are showing increased abilities in determining the structure of small membrane proteins with similar molecular weights. Glyco-diosgenin (GDN) has been successfully used in a number of recent cryo-EM studies of membrane proteins as an effective drop-in substitute for digitonin [31,32]. Therefore, after the C-terminal Twin-Strep tagged hDHHS3-Δ35 was expressed in High Five cells and solubilized by LMNG, the membrane protein crude mixture was loaded onto the Strep-Tactin resin and then subjected to the detergent exchange from LMNG to GDN, which was followed by size exclusion chromatography (Figure S5B). The fractions from the elution peak at 10.44 mL (indicated as “1” in Figure 4A) were collected for cryo-EM using a Talos Arctica microscope.

Although further 3D classification revealed no detailed information, the original micrograph and the 2D classification thereof showed an even-distributed homogenous particles in samples with a typical sideview of membrane proteins (Figure 4C). Most of these classes had ellipsoidal shapes with an extra-membranous domain protruding out from the micelles, suggesting the potential of the EM samples of hDHHS3-Δ35 in structural solving upon further optimizations on sample preparation and electronic microscopic methods.

## 4. Discussion

In this study, we first described the expression and purification of hDHHC3 and obtained a homogeneous preparation of hDHHC3 for crystallization tests. The disordered NTD was removed to avoid the degradation during the purification, which decreased the inherent heterogeneity substantially. C157S mutation and 2-BP inhibitor were also examined for the crystallization. However, no crystals were obtained at any tested crystallization conditions. We further identified the interacting proteins of hDHHC3 by mass spectrometry to increase the crystallization possibility. Several proteins can be pulled down together with hDHHC3, including LAMTOR1, JAGN1, TMX3, etc. LAMTOR1 was further investigated due to its palmitoylation reported previously. The crystallization trials failed to be executed due to the unsuccessful expression of LAMTOR1, since LAMTOR1 usually acts as a scaffold protein which requires other proteins to assist its expression [33]. On the other hand, whether DHHC3 is the specific palmitoyltransferase for LAMTOR1 deserves further investigations. Recently, Lee et al. indicated that the addition of palmitoyl-CoA to an inactive mutant of DHHC20 (hDHHS20) can be successfully crystallized [24]. This crystal structure revealed that palmitoyl-CoA locates at the interface between two hDHHS20 monomers and partially inserts into the acyl chain binding cavity, which gives the clue to improve crystallization conditions to obtain the DHHC3 structure at atomic resolution.

We noticed that a dimeric state exists in the 2D classes of the inactive hDHHS3 from the cryo-EM studies, indicating a dynamic balance between the monomers and dimers maintaining in the protein samples. This heterogeneity observed in the cryo-EM analysis provides one explanation of the difficulty for crystallization of the inactive hDHHS3. In addition, the deep buried transmembrane helices and lack of a large soluble segment for observations make the cryo-EM study even more challenging for the small membrane protein as hDHHC3. Previously, it has been shown that binding of the target protein to nanobodies increases the overall protein size such that sufficient contrast and features can be obtained [34]. This would be an alternative strategy to overcome the low size barrier for cryo-EM structure determination. Therefore, further investigations on obtaining nanobodies could be considered in the subsequent study. 

DHHC3 catalyzes S-palmitoylation in two steps. In the first step, the palmitoyl is added to the catalytic cysteine. In a second step, the palmitoyl group is transferred onto a target protein in a transpalmitoylation reaction [35]. Our results from the coupled enzyme assay showed that NTD-truncated hDHHC3s retain the auto-palmitoylation activity; i.e., the first step of the reaction occurs, indicating that the NTD is not essential for the first step but related to the palmitoyl group transferring in the second step. However, NTD-truncated hDHHC3s showed smaller K_m_ values, inferring to the higher binding affinity to palmitoyl-CoA, which implies that the substrate binding is self-sequestered by the endogenous hDHHC3-NTD. Meanwhile, the AlphaFold2 prediction of DHHC3 showed a very similar architecture to the crystal structures of DHHC15 and DHHC20 especially at the transmembrane domain, although the sequence identity is low. The large differences between DHHC3 with DHHC15 or DHHC20 were observed on the NTD—that DHHC3 has an extended N-terminal region with high flexibility and dynamics. The structural flexibility of the NTD might explain why DHHC3 can target a broader range of proteins [36]. Elucidation of the DHHC recognition of the target proteins at the atomic level requires further efforts.

## Figures and Tables

**Table 1:** Proteins identified by the pull-down assay combined with LC-MS/MS analysis.

Description				Coverage (%)	Peptides	PSMs ^b^	Unique Peptides
Protein Name	Species ID	Gene Name	Sum PEP Score ^a^				
Palmitoyltransferase ZDHHC3(bait)	9606	ZDHHC3	119.303	47	15	172	15
Synaptojanin-2-binding protein	9606	SYNJ2BP	33.322	84	8	10	8
Peroxiredoxin-5	9606	PRDX5	24.54	37	6	7	6
Pinin	9606	PNN	12.737	10	6	6	6
Syntaxin-12	9606	STX12	20.493	24	5	6	5
ATP-binding cassette subfamily F member 1	9606	ABCF1	16.657	9	6	6	6
Protein disulfide-isomerase TMX3	9606	TMX3	13.717	15	5	6	5
Protein jagunal homolog 1	9606	JAGN1	11.936	13	2	5	2
S-methyl-5′-thioadenosine phosphorylase	9606	MTAP	13.341	25	5	5	5
Cystatin-B	9606	CSTB	20.6	46	3	5	3
Mannose-6-phosphate isomerase	9606	MPI	17.188	17	4	5	4
Phosphoserine aminotransferase	9606	PSAT1	38.293	32	12	13	12
Sodium/potassium-transporting ATPase subunit beta-3	9606	ATP1B3	25.731	19	4	8	4
Adipocyte plasma membrane-associated protein	9606	APMAP	36.867	36	11	12	11
Ragulator complex protein LAMTOR1	9606	LAMTOR1	16.821	40	4	4	4
Aldehyde dehydrogenase	9606	ALDH2	21.245	23	7	8	7

^a^ Sum PEP Score: sum of negative logarithms of posterior error probability (PEP) values for all peptide spectrum matches (PSMs ^b^). The higher the value, the higher the credibility. PSMs indicate the total number of identified peptide sequences matched for the protein.

## Data Availability

Not applicable.

## Supplementary Materials

The following supporting information can be downloaded at: https://www.mdpi.com/article/10.3390/polym14153013/s1, Figure S1: Sequence alignment of DHHC3 homologs; Figure S2: Sequence alignment of DHHC3, DHHC15 and DHHC20; Figure S3: Expression and purification of truncated hDHHC3s; Figure S4: Detergent screening of hDHHC3-Δ21; Figure S5: Dimerization of hDHHC3-Δ35 and hDHHS3-Δ35; Table S1: Sequence identity matrix of mammalian DHHC3. Reference [37] is cited in the supplementary materials.
