# Supplementary material for: Structural Exploration on Palmitoyltransferase DHHC3 from Homo sapiens
Source: Polymers (Basel). 2022 Jul 26;14(15):3013. doi: 10.3390/polym14153013 (PMC9332573; doi:10.3390/polym14153013)
Supplement: Supplementary file 1 [file polymers-14-03013-s001.zip › polymers-1805871-supplementary.pdf]

polymers

MDPI

Article

Structural exploration on palmitoyltransferase DHHC3 from *Homo sapiens*

Meng Tang [1,2], Ying Xia [3,4], Taoran Xiao [1,2], Ruiyu Cao [1,2], Yu Cao [3,4,*] and Bo OuYang [1,2,*]

1 State Key Laboratory of Molecular Biology, Centre for Excellence in Molecular Cell Science, Shanghai Institute of Biochemistry and Cell Biology, Chinese Academy of Sciences, 320 Yueyang Road, Shanghai 200031, China; tangmeng6@sibcb.ac.cn (M.T.); xiaotaoran2018@sibcb.ac.cn (T.X.); caoruiyu2018@sibcb.ac.cn (R.C.)
2 University of Chinese Academy of Sciences, Beijing 100049, China
3 Institute of Precision Medicine, The Ninth People's Hospital, Shanghai Jiao Tong University School of Medicine, 115 Jinzun Road, Shanghai 200125, China; xiaying@shsmu.edu.cn (Y.X)
4 Department of Orthopaedics, Shanghai Key Laboratory of Orthopaedic Implant, Shanghai Ninth People's Hospital, Shanghai Jiao Tong University School of Medicine, Shanghai 200011, China
* Correspondence: yu.cao@shsmu.edu.cn (Y.C.); ouyang@sibcb.ac.cn (B.O.)

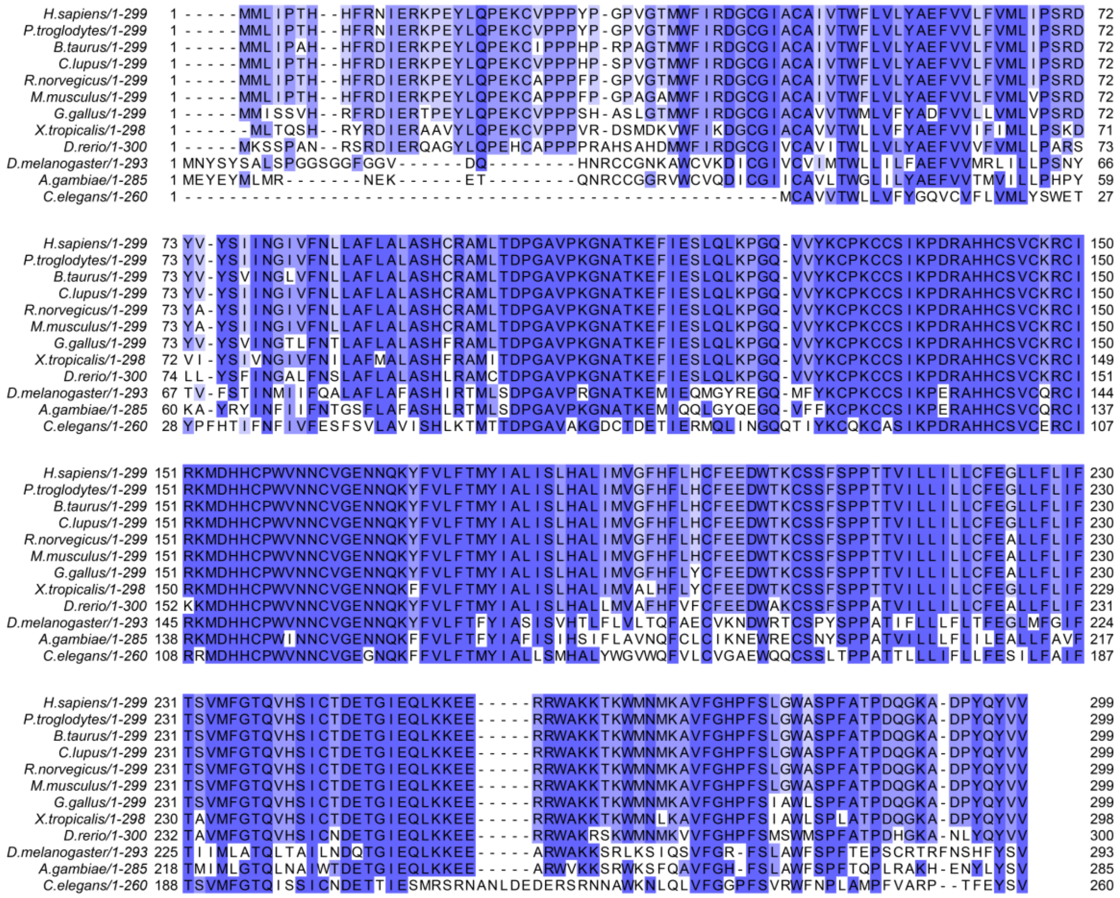

Figure S1. Sequence alignment of DHHC3 homologs. Amino acid sequences of DHHC3 homologs were aligned by Clustal Omega [37]. Species contain *Homo sapiens* (human), *Pan troglodytes* (chimpanzee), *Bos taurus* (bovine), *Canis lupus* (wolf), *Rattus norvegicus* (rat), *Mus musculus* (mouse), *Gallus gallus* (Chicken), *Xenopus tropicalis* (clawed frog,), *Danio rerio* (zebrafish), *Drosophila melanogaster* (fruit fly), *Anopheles gambiae* (mosquito), *Caenorhabditis elegans*.

Table S1. Sequence identity matrix of mammalian DHHC3

	H. sapiens	*P. troglodytes*	*C. lupus*	*R. norvegicus*	*M. musculus*	*B. taurus*
Homo sapiens	100	100	99	98.33	97.32	97.32
Pan troglodytes	100	100	99	98.33	97.32	97.32
Canis lupus	99	99	100	98.33	97.32	97.99
Rattus norvegicus	98.33	98.33	98.33	100	99	96.99
Mus musculus	97.32	97.32	97.32	99	100	96.66
Bos taurus	97.32	97.32	97.99	96.99	96.66	100

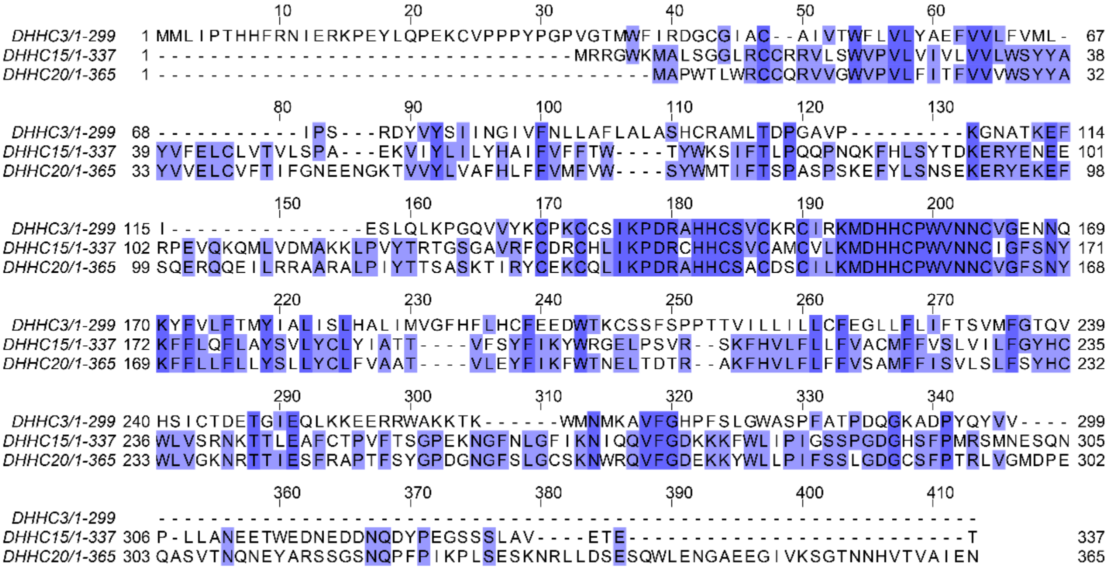

Figure S2. Sequence alignment of DHHC3, DHHC15 and DHHC20. Amino acid sequences of human DHHC3, DHHC15 and DHHC20 were aligned by Clustal Omega [37]. DHHC3 shows low sequence identity with DHHC15 (25.4 %) and DHHC20 (29.5 %), while CRD including DHHC motif is highly conserved in these 3 members.

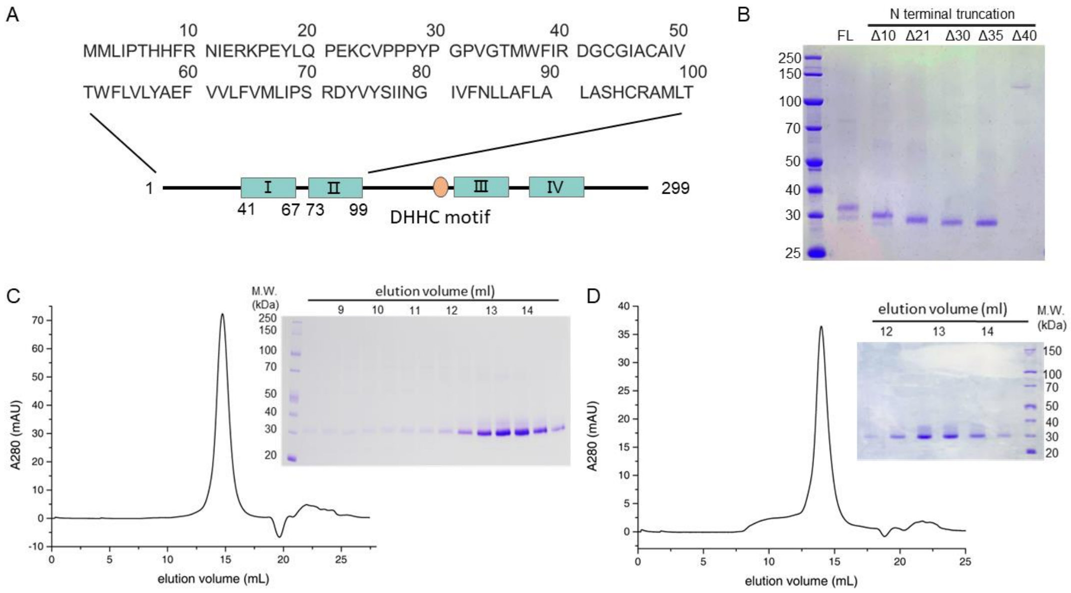

Figure S3. Expression and purification of truncated hDHHC3s. (A) Topological scheme and N-terminal sequence of hDHHC3. (B) Expression test of hDHHC3 with different N-terminal truncations. (C) Size exclusion chromatography and SDS-PAGE of hDHHC3-Δ21 and (D) hDHHC3-Δ35.

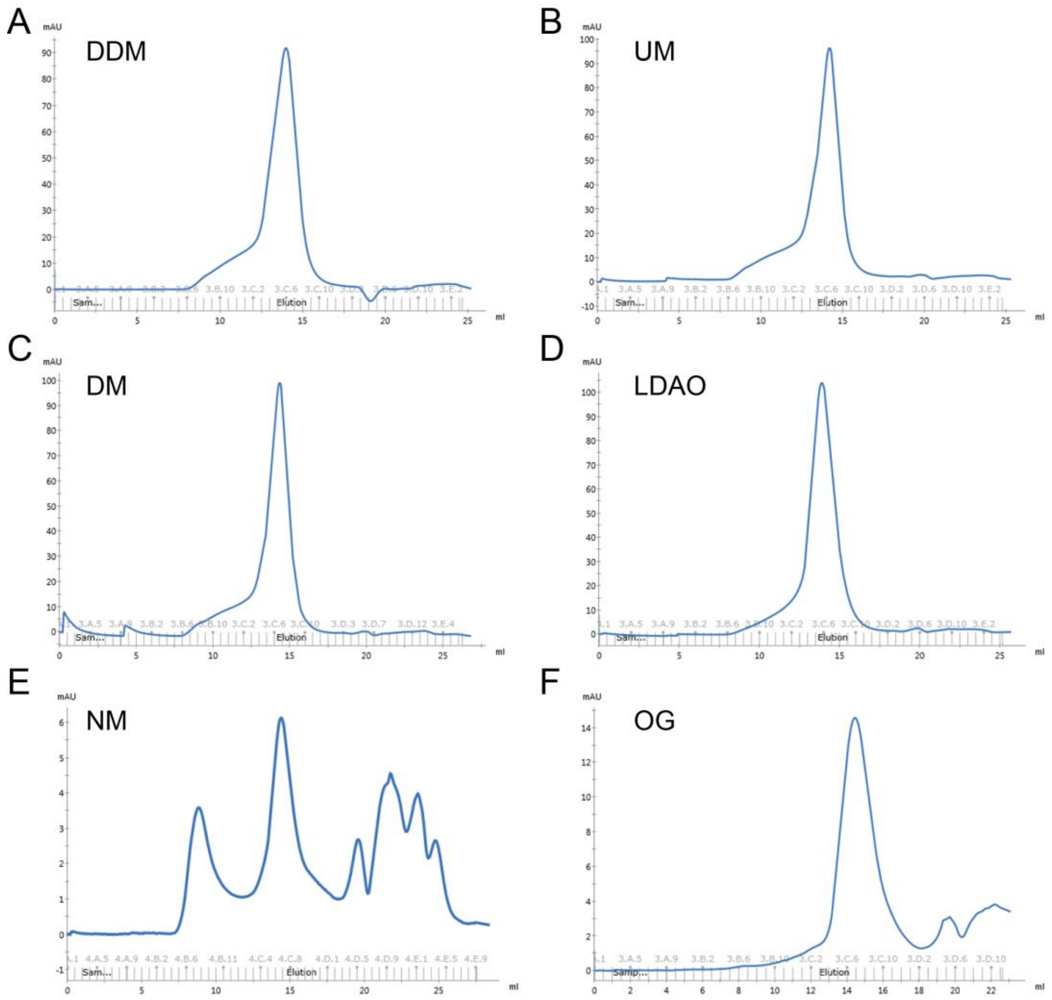

Figure S4. Detergent screening of hDHHC3-Δ21. Size exclusion chromatography profiles of hDHHC3-Δ21 in FPLC buffer containing 25 mM HEPES, 150 mM NaCl with (A) 1 mM DDM, (B) 2 mM UM, (C) 4 mM DM, (D) 0.15 % LDAO, (E) 12 mM NM and (F) 40 mM OG, respectively.

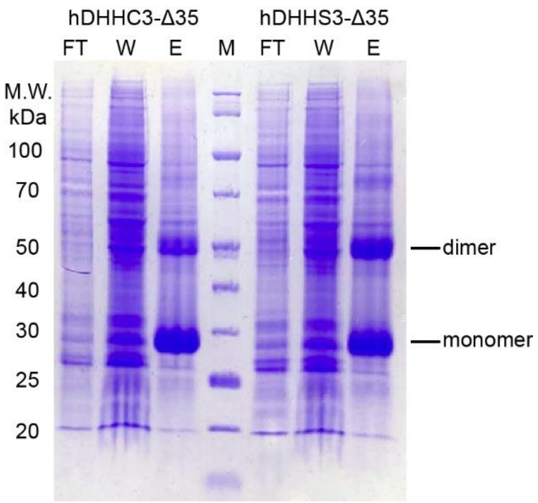

Figure S5. Dimerization of hDHHC3-Δ35 and hDHHS3-Δ35. An obivous dimeric band of hDHHS3-Δ35 was observed on the SDS-PAGE gel.

Reference

37. Madeira, F.; Pearce, M.; Tivey, A.R.N.; Basutkar, P.; Lee, J.; Edbali, O.; Madhusoodanan, N.; Kolesnikov, A.; Lopez, R. Search and sequence analysis tools services from EMBL-EBI in 2022. *Nucleic Acids Res.* **2022**, doi:10.1093/nar/gkac240.
